# Future implications of polygenic risk scores for life insurance underwriting

**DOI:** 10.1038/s41525-024-00407-x

**Published:** 2024-03-30

**Authors:** Tatiane Yanes, Jane Tiller, Casey M. Haining, Courtney Wallingford, Margaret Otlowski, Louise Keogh, Aideen McInerney-Leo, Paul Lacaze

**Affiliations:** 1 Frazer Institute, The University of Queensland, Dermatology Research Centre, Brisbane, QLD Australia; 2 Department of Epidemiology and Preventive Medicine, School of Public Health and Preventive Medicine, Monash University, Melbourne, Australia; 3 Centre for Health Equity, Melbourne School of Population and Global Health, University of Melbourne, Victoria, Australia; 4 Centre for Law and Genetics, Faculty of Law, University of Tasmania, Churchill Avenue, Hobart, Tasmania Australia

**Keywords:** Genomics, Health policy

The use of genetic risk information in life insurance underwriting is a major ethical, legal, and psychosocial concern^1–4^. Genetic discrimination (GD) is defined as the “*differential treatment of asymptomatic individuals or their relatives on the basis of real or assumed genetic differences or characteristics*”^3^. In life insurance underwriting, GD stems from the use of genetic risk information to deny coverage, increase premiums, or place conditions on products such as disability, death, trauma and income protection cover^5^. Reports of insurance discrimination among individuals with rare variants in monogenic risk genes are well described, including insurance providers denying coverage or increasing premiums based on positive results, even when individuals take steps to mitigate risk^5–9^. However, it is difficult to quantify the prevalence of GD due to methodological challenges such as ability to obtain data from insurance industry, thus, most research to date has relied on self-reported experiences. Nevertheless, fear of GD remains a deterrent to uptake of genetic testing in clinical and research settings^6–14^. With the emergence and increased use of new genetic technologies it is essential that we consider the unique challenges that may arise regarding GD.

An emerging genomic technology that is likely to present new challenges in GD is polygenic scores (PGS). PGS provides an estimate of the genetic liability to health conditions and is typically calculated based on the cumulative impact of multiple disease-associated common genetic variants or single nucleotide polymorphisms (SNPs), derived from genome-wide association studies (GWAS)^15,16^. Several articles have considered the future clinical implications of PGS^17–22^, but few have considered insurance implications specifically^23^. PGS has the potential to disrupt the insurance industry given its broad use in risk-stratification for common complex health conditions^17^. Furthermore, there is emerging evidence that the risk of insurance discrimination may negatively impact willingness to undertake PGS testing and participate in research^24^. Thus, the increased use of PGS and its possible impact on life insurance underwriting warrants further consideration. In this article, we explore the current measures to address GD in insurance underwriting globally, issues of GD arising from PGS use, and argue that the increased availability of PGS could shift the way insurers utilize genetic risk information. As life insurance is the risk-rated product that has been most frequently evaluated in the context of GD it is the focus of this article.

## International measures to address genetic discrimination in insurance

Box 1 provides a definition of community vs risk-rated insurance, which provides important context in understanding the impact of genetic information in insurance underwriting. Internationally, various regulatory measures have been introduced to address GD in insurance underwriting^2,25–29^ (see Table 1 for some examples). These measures range from soft forms of regulation such as industry-led moratoria (e.g., Australia)^26^ and voluntary agreements between governments and industry (e.g., UK)^25^, to relatively more robust regulatory responses in the form of legislation as found in Canada^27^. In addition to the variation in the type of regulation, the scope of the protection each regime offers varies. For instance, some protections only apply to certain types of insurance (e.g., the federal US protection extends only to health insurance and employment, not life insurance)^28^, and others only apply within prescribed financial limits (e.g., Australia’s moratorium). Other countries, such as New Zealand, currently do not have any protections against the use of genetic information in health or life insurance^8^. The Australian Government recently recognized the level of community concern in Australia about genetic discrimination in life insurance^30^, and conducted a consultation on options to address the issue (concluded 31 January 2024)^31^, which received over 1000 stakeholder submissions to Treasury (Tiller J., Personal Communication Treasury Department, Feb 06, 2024).

**Table 1:** Overview of genetic discrimination protective regimes for Australia, Canada, USA, and United Kingdom and potential applicability to PGS

Region/country and name of protection	Scope of protection	Limits	Applicability to PGS
Australia FSC Standard 11: Moratorium on Genetic Tests in Life Insurance^12^	Industry led moratorium that applies to individually underwritten life insurance. This does not extend to private health insurance, which is community rated (i.e., premiums apply universally) and not subject to underwriting. Under the moratorium, insurance providers can ask for or use **genetic test** results if the total amounts of cover would exceed the prescribed **limits**, provided that an evidence base shows the test has relevance to the cover applied for. Favorable Genetic Test results and evidence based preventative treatment, or adherence to evidence based preventative measures, can be accounted for in underwriting. Underwriters cannot ask applicants to get genetic testing or disclose genetic test results taken as part of a medical research study (provided the applicant has not received the results or asked not to receive them).	•$500,000 Death Cover. •$500,000 Total permanent disability Cover •$200,000 Trauma and/or critical illness Cover. •$4,000/ month Income protection	**Genetic test** is broadly defined as ‘a test which examines a person’s chromosomes or DNA’ but does not include non-genetic medical tests. The current framing is likely broad enough to capture PGS. For genetic tests to be used in underwriting the limits must be exceeded and insurers need to justify that the test has relevance to the cover being applied for (per *Disability Discrimination Act 1992* (Cth)).
Canada: Genetic Non-Discrimination Act^13^	Federal legislation that prohibits any person from requiring an individual to undergo a **genetic test** or provide the results of a **genetic test** as a condition of: •providing goods or services to that individual •entering into or continuing a contract or agreement •offering or continuing specific terms or conditions in a contract or agreement.	No limits	**Genetic test** is broadly defined as ‘a test that analyzes DNA, RNA or chromosomes for purposes such as the prediction of disease or vertical transmission risks, or monitoring, diagnosis or prognosis’. The current framing is likely broad enough to capture PGS.
UK: Code on Genetic Testing and Insurance^11^	Agreement developed between the UK Government and Association of British Insurers (ABI) which applies to ABI members. Insurers will not require applicants to undertake a diagnostic or predictive **genetic test** to obtain insurance and will only ask applicants to disclose the result of a predictive **genetic test** result for *approved* conditions (currently only Huntington’s disease) for policies above the prescribed financial limits. For insurance other than life, critical illness and income protection, predictive genetic test results will not be asked for, or taken into account, whatever the level of cover. Genetic tests results given to the insurer accidentally or voluntarily will only be considered if it is for the applicant’s benefit.	Life Insurance £500,000 Critical Illness £300,000 Insurance Income Protection £30,000	**Genetic test** ‘refers to a test which looks for a particular gene variant. This is regardless of whether the test was carried out as part of a single-gene test, a panel, or up to the level of whole genome sequencing’. The current framing is likely broad enough to capture PGS.
US: Genetic Information Nondiscrimination Act (GINA) and state laws^14^	The federal protection extends only to health insurance and employment, it sets out the base level of protection with additional protections left to the discretion of individual states. Some states have enacted laws providing consumers additional protections in the context of life insurance, but protections are often limited. Indeed, most states enacting additional protective legislation subject the use of genetic information to informed consent or actuarial requirements rather than outright prohibition.	Protection only extends to health insurance and employment, not life insurance. Does not apply to employers with fewer than 15 employees.	Under GINA, **genetic test** means ‘an analysis of human DNA, RNA, chromosomes, proteins or metabolites, that detects genotypes, mutations or chromosomal changes’. The current framing is likely broad enough to capture PGS. With respect to state laws, definitions of genetic test are determined by individual state legislation but similarly tend to rely on broad definitions.

Box 1 Important context for understanding insurance products Insurance products are either community-rated or risk-rated^55^.

- Community rated: each consumer is charged the same premiums. In many countries, health insurance is community rated.
- Risk-Rated: differentiation of premiums based on individual consumer risk assessment, which can include genetic risk information. Typically applies for life, disability, long-term care, and travel insurance.

## Polygenic scores (PGS) in clinical practice

PGS is best considered as a risk-stratification or screening tool rather than diagnostic, and it can be used to predict the possibility of health conditions or behavioral traits. There are various reported uses of PGS that include informing population screening programs for common complex conditions, such as cancers, heart disease and diabetes^17^. Testing for PGS can also be used to inform treatment and risk management strategies, predict diagnostic outcomes, and modify risk for monogenic conditions^17^. Given the broad use of PGS, it is important to consider the context in which the information is being used. For example, the predictive ability of the PGS is bounded by the heritability of the condition of interest, and therefore may be less useful for conditions with low heritability^32^. Furthermore, PGS estimates are calculated based on data derived from GWAS. Currently, >80% of GWAS data has been obtained from European populations, thereby limiting the predictive performance of PGS to non-European populations^32,33^. There is strong evidence for the clinical validity of PGS (i.e., the test’s ability to accurately and consistently predict outcomes of interest), while clinical utility is yet to be determined (i.e., the test’s ability to improve health outcomes)^32,34^. Nevertheless, consumers are increasingly accessing PGS testing through direct-to-consumer companies and third-party providers^35,36^, clinical research^37^, and commercial genetic testing companies^17,18^.

Implementation of PGS has the capacity to change the way insurers consider and use genetic information. The life insurance industry is already aware of the potential impact of PGS in healthcare and has identified PGS implementation as a possible challenge for the insurance industry^38,39^. Specifically, industry commentators have noted the increased use of genetic testing in the population, and have proposed potential solutions, such as applying a community rating structure where assessments are pooled to support claims for conditions that have a high genetic burden, rather than using an individual risk-rated approach to underwriting^39^. Additionally, using an aggregate PGS for 27 common conditions in an elderly population, Linnér et al.^23^, reported a 2.6-year shorter median lifespan in the highest decile group and proposed that this data could be used to improve mortality risk classification in life insurance. However, mortality estimates are complex and not easily explained by PGS. Early research suggested PGS have a fairly moderate predictive capacity, and that a substation proportion of the associated risk is accounted by common mortality risk factors already measured in middle age^40,41^.

## Considerations of PGS and life insurance underwriting

### Increased accessibility of genetic risk assessments

Traditionally, genetic testing has been used to identify the <5% of the general global population suspected to have a rare monogenic condition^42,43^. Guidelines for monogenic testing vary between countries, organizations, and conditions^44–46^. However, most criteria for publicly funded genetic testing (or testing through insurance providers) include risk assessments to identify those most likely to carry pathogenic variants in disease risk genes. Only a small portion of those at risk for developing a condition are targeted for genetic testing, limiting the number of individuals whose genetic test results might then be used in life insurance underwriting. Conversely, PGS have much broader clinical application (e.g., population screening programs, and augment monogenic testing^17^) and can be developed for most health conditions and heritable traits (such as obesity^47^). Widespread implementation of PGS will result in genetic risk assessments accessible to most of the population across various settings, potentially amplifying GD in insurance underwriting.

Current GD protections tend to apply to use of ‘genetic tests’ (Table 1), which is broadly defined in the various protective regimes (e.g., tests that examine chromosomes and DNA). Some commentators have argued that the broadness of this definition makes it unclear what types of genetic testing (and hence protection) are captured^48^. It is possible, in the absence of guidance to the contrary, that current protections may extend to PGS. However, the current lack of clarity is undesirable given that PGS has the potential to increase the volume and diversity of genetic results available to insurers. If no additional consumer protections are introduced, there is a danger that PGS will amplify the risk and frequency of GD in life insurance underwriting.

### PGS as a nascent risk prediction tool

Despite commercial availability, there are currently no best practice guidelines for developing and reporting PGS, and evidence for clinical utility is still emerging^15,16,32^. Several professional organizations have released position statements on the use of PGS in clinical practice, which commonly acknowledge the potential benefits of PGS, while urging for caution given the limited evidence for its clinical utility^49–51^. Statistical methods for calculating PGS are constantly being improved and new GWAS data is being generated. The lack of ancestry diversity in GWAS, resulting in reduced predictive performance of PGS in non-European populations, is widely recognized as a major limitation of PGS^33^. As such, an individual’s PGS today may differ from one calculated in the future due to changes to the methodology, new GWAS data, and improvements in ancestry data, which could result in different risk classifications and altered medical advice for individuals^52^.

A PGS is a standalone risk factor, which does not typically consider the impact of rare monogenic variants or clinical and lifestyle risk factors^16^. To account for additional risk factors, PGS is being integrated into comprehensive risk assessment models, such as the CanRisk tool that provides personalized breast cancer and ovarian risk based on monogenic, polygenic, family history, clinical and lifestyle factors^53,54^. Such complex risk prediction tools increase the likelihood of risk estimates changing over time. Importantly, these tools reflect the reality that PGS is not diagnostic information. There is a real concern that insurance providers will seize the opportunity to use PGS alone to classify a person’s risk and exclude individuals they consider “high risk”, without considering the remaining dynamic risk factors. Lastly, it is important to note that no one person will have a low genetic risk for all possible health conditions and traits, and it is not known how different conditions and traits would be weighted by life insurance providers.

### Potential for misinterpretation

Given the nascent state of PGS, there is significant potential for misinterpretation and misuse of the PGS information by life insurance providers (Box 2). Despite monogenic testing being available for more than 25 years, there is evidence that insurance providers still misinterpret results and have failed to consider the impact of risk-reduction strategies in underwriting^5,55–57^. Compared to monogenic testing, a PGS is substantially more complex, and interpretation requires comprehension of genetic and epidemiological concepts. Aspects of PGS that have the potential for misinterpretation include failure to appreciate the risk assessment nature of PGS, its limitations for non-European populations, and limited predictive ability across family members (Box 1). Research has shown that even genetics professionals currently struggle to interpret and explain PGS given the lack of existing education and clinical guidelines for this test^58,59^. As such, it is anticipated that insurance underwriters would also have difficulties interpreting and using this information in risk assessment. As all stakeholders are unlikely to understand the nuances of a PGS, especially in the early days of implementation, careful consideration needs to be given to how risk information is delivered to mitigate both the potential for insurance provider misinterpretation and exacerbation of GD in life insurance^20^.

Box 2 Potential areas for misunderstanding and misuse of PGS information by insurance providers

- **Misunderstanding PGS as a diagnostic tool, rather than risk stratification:** Insurance providers may fail to understand PGS as a screening tool, whereby the degree in which the PGS predicts disease risk is based on various factors, such as disease/trait heritability, background population risk, the statistical methodology used to generate the PGS, and impact of other genetic and non-genetic risk factors^16,17,19^. Despite insurance underwriters’ expertise in assessing risk factors and conducting complex risk assessments, concern remains regarding the scientific and medical complexity of PGS and the ability of underwriters to interpret PGS without specialist training.
- **Failure to appreciate limitations of PGS across diverse populations:** The validity of PGS is inherently dependent on the quality of the GWAS data on which it was based. Currently, greater than 80% of GWAS data has been obtained from populations of European ancestry^68^, resulting in PGS that have reduced predictive performance in individuals from other ancestries. Insurance providers may fail to consider the impact of ancestry on PGS, which in turn may compromise the accuracy of their risk estimates.
- **Misinterpretation of risk for family members:** Genetic testing has traditionally been considered within a familial context, with Mendelian inherence patterns used to estimate risk to relatives. Further, insurance underwriting models also use genetic information, including a family history of disease as a predictor of risk for related individuals. However, current PGS are personalized and cannot be used to estimate risk for close relatives’ results. While there is some association between siblings PGS, this relationship and predictive capacity becomes weak for parents and 2nd degree relatives^69,70^. It is possible insurance companies will interpret familial risk based on PGS, potentially leading to further insurance discrimination.

### Arguments by insurance companies

The insurance industry commonly raises concerns about adverse selection and the impact of risk prediction on the affordability of insurance^60,61^. When the Canadian Genetic Nondiscrimination Act 2017 was being considered, the Canadian Privacy Commissioner commissioned two statistical experts to conduct modeling to consider the potential impact of banning the use of genetic test results in life and health insurance^62,63^. Both found that the impact of a ban on the insurance market in the medium term would be negligible. No modeling was conducted at the time regarding the impact of PGS on insurance affordability, and such studies would be worthwhile. We anticipate that although PGS would be relevant to the entire population, the lower predictive value relative to monogenic tests means that the results are not deterministic, and thus, the impact on the market is not likely to be substantial^17^.

Arguments about adverse selection become less significant when considering population-level risk stratification. Adverse selection refers to the notion that people at higher risk will take out more expensive policies, therefore skewing the affordability of insurance for all^64^. However, if PGS is used as a population-level risk stratification tool, every person in the population is likely to have higher PGS for some disease types and lower PGS for others. Furthermore, ethically, we note that insurance is supposed to be a risk-pooling exercise, not an exercise in eliminating high-risk individuals from the risk pool^65^.

### Moving forward

As PGS is increasingly utilized in research and clinical practice, it is pivotal that careful consideration is given to the potential insurance implications of PGS to ensure consumer protection against GD. For the full potential benefits of PGS to be realized, and its clinical utility determined across various use cases, individuals will need to be confident that they can participate in research studies and access clinical genetic testing without fear of insurance discrimination. Clarification is needed regarding the extent to which existing protections and legislation relating to monogenic testing may also extend to PGS test results. Given there is little enforceable protection against GD in life insurance in various countries (Table 1) further legislative protection should be introduced, which clearly includes PGS in its protection. Additionally, clear guidelines, best practice protocols, and training are needed to support accurate interpretation of genetic risk information among insurance providers and minimize the risk of misinterpreting results. Finally, further research is needed to evaluate future issues of GD arising from PGS implementation.

### Recommendations

The ethical, legal, and social issues described above highlight a pressing need for improved consumer protection, and improved implementation research to support the equitable implementation of PGS into clinical practice. In our view, the use of PGS as a tool to further deny coverage is both ethically questionable and actuarially problematic. We recommend that a ban on the use of PGS results in risk-rated insurance underwriting should be introduced. This recommendation stands alongside calls to prohibit the use of genetic test results more broadly in life insurance underwriting, which authors of this paper and others have made^66^. Consideration should be given to the most appropriate regulatory tools to achieve this end in each jurisdiction, given the human genetics field is rapidly evolving^67^. At a minimum, it is crucial that:

- any regulation creates enforceable remedies for individuals and is subject to independent oversight by a body with meaningful sanction powers;
- any regulation has sufficient flexibility to respond adequately to advances in the field of genetics;
- all current regulations/consumer protections explicitly apply to both monogenic testing and PGS (or are amended to provide protection where it is determined that they do not apply); and
- insurers are educated about the limitations of PGS as risk prediction tools.
